# From Being Caregiver to Being Cared for: The Experience of Adapting to Spinal Cord Injury, a Case Study

**DOI:** 10.3390/nursrep12030054

**Published:** 2022-08-05

**Authors:** Monserrat Fernández-Moya, Marcela Ortega-Jiménez, María Elisa Moreno-Fergusson

**Affiliations:** 1 Life Sciences Division, Nursing and Obstetrics Department, Campus Irapuato-Salamanca, University of Guanajuato, Guanajuato 38140, Mexico; 2 Nursing and Obstetrics Department, Celaya-Salvatierra Campus, University of Guanajuato, Guanajuato 38140, Mexico; 3 Doctoral Program in Nursing, Faculty of Nursing and Nutrition, University of La Sabana, Chía 53753, Colombia

**Keywords:** spinal cord injury, Ricoeur, adaptation, chronic conditions

## Abstract

This narrative case study portrays a young woman’s life experience and adjustment process after suffering a traumatic spinal cord injury (SCI) 5 years ago. It is analyzed retrospectively from the perspective of the middle-range theory (MRT) of adapting to chronic health conditions by Buckner and Hayden (2014), and Ricoeur’s narrative philosophy is expanded. Understanding Alice’s narrative from this perspective allows us to understand the process of adaptation to a condition of disability due to a spinal cord injury, from the perspective of a nurse who was forced to change her role as a caregiver to a role of being cared for, due to the changes in her body and her corporality due to the consequences of the injury. In this narrative, the focal and contextual stimuli, the coping processes with special emphasis on the intrinsic and extrinsic adaptive processes, and the results of the process are identified.

## 1. Introduction

In this case study in narrative, a retrospective analysis is made of the experiences of Alice, a woman who, at the age of 34, suffers a car accident, as a consequence of which she experiences spinal cord injury at the level of the T11–T12 vertebrae. Spinal cord injury (SCI) is a term used to refer to damage to the spinal cord as a result of trauma. There are no reliable statistics on its prevalence, however, it is estimated that the global incidence ranges between 40 and 80 cases per million inhabitants; of these, up to 90% originate from traumatic causes [1].

The person who suffers a SCI has various sequelae and complications derived from the changes they have to face [2,3]; The sequelae include paralysis and altered sensitivity below the level of the injury, loss of sphincter control, autonomic nervous system disorders, and alterations in sexual activity and fertility [4]. These sequelae cause loss of autonomy, because they have to depend on a caregiver to be able to carry out activities of daily living [5] and on medical devices and accessories that facilitate their mobility and independence [2].

Regarding mental health, patients with SCI have an elevated risk of presenting anxiety or depression after hospital discharge, especially among younger patients (<50 years), compared to groups with other health conditions [3]. Some evidence shows that the difficulties experienced by people with SCI after hospital discharge are loneliness and isolation after hospital discharge, the perception of lack of physical, practical, and psychological support, and negative and stigmatizing reactions from the community in general [6]. People with SCI are forced to relearn a new way of conducting their activities, and to adapt the physical environment where they develop [7], so that it is accessible.

This study is presented with a narrative approach, this approach has been incorporated in recent decades in the production of knowledge in qualitative research in health, in the understanding of the sick subject, providing clues about the ways in which the body, self, and society are linked in late modern cultures [8].

Castellanos argues that narratives not only organize interpretations, but also consist of a specific form of social agency. In this sense, narrative interpretations can be seen as central elements of the social construction of experiences and trajectories of illness and care [9]. For his part, Good [10] mentions that narratives are stories that change as events unfold, point to the future with hope and anxiety, and often support various provisional readings of the past and present, trying to make sense of the experience.

Although narrative research is interdisciplinary, often the interests of the disciplines tend to take specific approaches. Denzin [11] points out five fundamental approaches to contemporary narrative research, within which are the narrative of psychologists and life history; sociologists and identity work; another sociological approach bases their interviews on specific aspects of people; the fourth approach, known as narrative ethnography, corresponds to the anthropologists; and finally, the autoethnography, whereby the researchers interpret their own narratives.

In this study, the particular experiences of the participant are extended into an integral narrative, exploring how living the SCI experience influenced her; these experiences are analyzed from the perspective of Buckner’s middle-range theory of adapting to chronic health conditions. To do this, we rely on the phenomenology of sensation and time of Paul Ricoeur’s Philosophy of Time; identity narration contributes to our understanding of how SCI generates changes in a person’s life.

## 2. Materials and Methods

The narrative approach places the people studied at the center of the study process and privileges the meanings they assign to their stories; listening to others´ experiences can be a source of support for people and can help them make informed decisions about their health. With the current emphasis on patient-centered care, research that focuses on the patient experience can also contribute to service delivery, design, and improvement [12]. The results attributed to the use of the narrative described in the literature are: the experience of chronic illness with an emphasis on the search for meaning, resignification of chronicity, and coping strategies; and the understanding of the disease from the perspective of the sick subject [13].

According to Ricoeur, we leave footprints when we express ourselves, and footprints give meaning to the world to which we belong. Often, the sense of footprints is hidden, making it impossible to directly understand the experiences of individuals. Reflection on an individual’s experiences must take place through the narratives in which the individual expresses himself [14]. In disease narratives, in particular, temporality is rarely linear. Disease by its very nature thwarts any pretense of temporal continuity, for it lacks the coherence that allows us to identify links between cause and effect, before and after. While it is true that any written or spoken narrative must start somewhere and end somewhere, the experience certainly has no clear time limits [15].

The information was collected in two moments, the first moment in an in-depth interview that lasted 2 h, in the second moment, the informant told her experiences in writing, in which she described the car accident, where the spinal cord injury was generated, her hospitalization, hospital discharge, incorporation into her daily life, and physical rehabilitation.

The interview was videotaped and transcribed. During the interview, the experiences reported from the past emerged in their present time as physical reactions, emotions, reminiscences, or memories. Sometimes past and present tense were confused. The informant also described situations in which she looked back and reflected on her past experiences, for example, the control and management of bowel and bladder dysfunction. All this is related retrospectively during the time in the interview. In narration, memories of past experiences were brought to her present time during reflection. As this material was analyzed, the distance in time increased even more. Thus, the material reflects how the narrated life intertwined between the experienced life and the speech, telling someone the experience, what Ricoeur describes in the three mimesis [16].

The first step of the analysis was the integration of the written narrative and the in-depth interview; the second step was the obtaining of codes and categories. The theoretical analysis was the last step. The MRT of adaptation to chronic health conditions is based on a body of descriptive and interventional research from multiple peer-reviewed studies [17]. The main concepts (categories) of stimuli, adaptation processes, and results of the participant’s experiences arise in relation to adaptation to spinal cord injury. For this reflection, it was necessary to identify the main concepts of midrange theory (TRM) of the chronic health conditions to later group the similar observations of the experiences reported by the informant during the interview and in the written narrative.

## 3. Results

Narrative

*For 34 years, my life passed without situations that tested my autonomy and my independence as a woman and as a person; I arrived on 14 May 2017, a day on which my whole life took a completely unexpected turn. I had several jobs, I taught at the university, and I was also working in a public hospital, that day I was going to my job as a nurse clinic, my work center was 45 min from the city road trip where I lived.*

*I was driving, thinking about what I would have to do, mentally preparing for the 12 h watch, when I unexpectedly lost control of my vehicle at a curve, the last thing I remember is taking the wheel of my car hard trying to regain control as the car passed from side to side across the road. No matter how hard I tried to control the vehicle, I couldn’t, and it started spinning down the road until I felt a strong impact. At that moment, I did nothing but put my hands to my head. All the light that was on that road went out.*

*I don’t remember with certainty what happened after, I was in a deep sleep from which I woke up for small periods, I knew I had an accident, I didn’t understand what was happening with my body, I didn’t feel the damage, I just knew I couldn’t move and I was inside my vehicle; I remember a man talking to me, he seemed to be between 50 and 60 years old, I remember he told me not to move, that the ambulance was coming; later, I remember paramedics talking to me, asking me where I worked, explaining that they would move me, and I lost consciousness again; I remember going in the ambulance and then hearing a voice asking “Is it Alice?”, at that moment I knew that I had already arrived; I was in the hospital where I worked; a colleague got into the ambulance and she asked me, how are you? And your daughter? I did not bring her, I left her with my father.*

*In the hospital I saw familiar faces, I remember being on the stretcher, what a strange feeling being in the place where you used to be, but now on the opposite side, being patient. All the health personnel were there, those with whom I always worked; there were those nurses who I directed for many years, with whom I talked, and those personnel, who for a long time, were my second family; some smiled, some others cried and in many others, I already saw them worried; but I felt safe, I knew I was in good hands, I knew that whatever they did to me, I would be fine.*

*I remember my body, I remember my rapid and ragged breaths, only my abdomen hurt, I didn’t feel anything else, I remember I told them my head bothered me in the occipital area, I felt the stretcher on my back, I knew they had me in protocol polytraumatized patient. They sent me for an X-ray, there was my partner, the one who always ran to help me move patients and with whom I shared innumerable talks in which I told him to continue encouraging his eldest son to finish nursing; then, I saw the radiology technician, I saw her surprised face, the nursing supervisor is not expected to take X-rays, he told me they would move me and that is all I remember of that moment, but they told me that that day my cries of pain were heard throughout the hospital, that it’s good that I don’t remember, it’s good that I forget that.*

*I heard murmurs, I heard the traumatologist say that he couldn’t assess or give a diagnosis if he did not have a tomography, even I did not understand, I did not even suspect. They began to find a place to take the study and they could not find it, I told them I have a medical network and there my great friend the cardiologist intervened, I heard him make a call and explain “if it’s an emergency, that’s it!” He said happily, they will receive her in Irapuato, we have to get an ambulance to take her immediately. I looked down and there was the director of the hospital and the head nurse, they couldn’t hide their worried faces, but there they were. Suddenly, I heard a how are you and it was my sister, she worked there too, only, on another shift, and behind her was my father, he didn’t speak to me, but his face also told me concern. The ambulance arrived, and the paramedic spoke to me, one of many with whom I was in the emergency corridors, he told me, “I’m going to take you to Irapuato, anything here I’ll go with you.”*

*They prepared me for transfer and there they were all, there was the one who used to be on my team, I saw them crying and very worried, I still didn’t understand what was happening, one of them told me, take care little girl, I told them I’m going to be fine; I love them very much.*

*We arrived in Irapuato, I saw my husband, I asked about my daughter and he told me that she was fine. I saw more familiar faces, professor friends. We arrived with the doctor, they turned me around so he could check me and in the middle of my back, I remember screaming a lot, he told me, “No, don’t move her anymore, let’s take her to the tomography”; I remember my husband telling me this, he is going to tell us everything. When he left the study I saw him differently, I saw him sad and worried, I listened to them making decisions, and they said, let’s go to León there is no neurosurgeon here; after this, I don’t remember much else, only ambulances, hospitals, and more doctors.*

*I remember a very nice doctor who was talking to my husband, he told him that they had to operate me if not the damage would be greater, I saw that they were talking but I did not understand; that day I accepted, I said that I wanted to be operated although I did not understand everything clearly, but I trusted that neurosurgeon. The days there were long, between doctor and nurses, until the day came when my neurosurgeon stood by my side, Alice you have fractured the vertebrae of the spine, he told me, fortunately, the spinal cord did not break, it was only damaged, and there I understood everything. I told him I will walk again and he remained silent, he told me that he had many patients who led their normal lives, that he had a teacher patient who had improved a lot. I still did not fully understand so that day I began to look for the diagnosis that my doctor had mentioned SCI.*

*A video platform was my greatest source of information, and there were people in wheelchairs, one video led me to another, they explained how they dressed, how they cleaned themselves, how some others did extreme sports, and how they drove. I kept assimilating what was happening to me; however, I did not want to feel defeated, in my mind I knew that I had to be strong, and I had to be fine because I thought of my little daughter.*

*They discharged me and there were more discoveries, I couldn’t sit up by myself and if I did, I would turn sideways, I didn’t have the strength to sit, so most of the time I was lying down. On the second day the visits started, friends, family, acquaintances, and students, everyone encouraged me and saw me as sick. My first achievement was done by myself, with the help of the bed rails, I managed to turn around, and I was happy because I no longer had to wake up anyone to get me moved, I knew that if I didn’t do it worse things would come. I knew that I shouldn’t spend so much time in one position; otherwise, I would develop bedsores, and that was my first achievement and there I tried to be independent again. I saw that I could if I tried it. The second achievement was to sit on the bed, I saw that my arms were now my strength and I liked it, again.*

*I loved being independent and doing things by myself, even if it was just to sit down; two weeks later they took me to the rehabilitation center, where they evaluated me and indicated three sessions per week. Thanks to the staff at the state rehabilitation center, I strengthened the muscles of my back, also those of my arms, little by little I advanced until I reached the full recovery of my autonomy and independence. I returned to the classroom, I needed to occupy my mind, being more active in my context gave me a new perspective, people with disabilities can develop in our environment; we must be present to be heard. Meanwhile, I continued to inform myself, and I saw the difficulties that many experience, the daily discrimination and not necessarily intentional discrimination, if not secondary discrimination, by not having an inclusive infrastructure. I saw everything that one goes through, but I learned not to remain silent, now I have taken that as a responsibility, and to every building that I go to that is not inclusive, I always want them to listen to me, make an observation of the areas of opportunity that they have, but more than anything, I seek to raise awareness, because I saw that by being in this condition, people around me live the experience with me; they even identify the places I could not enter because I am a wheelchair user.*

*I went back to my students; that day I was nervous, I was worried about the steps, but there I was again in the classroom, no, I would no longer be a clinical nurse, I would no longer take care of patients, now I would take care of students. I knew I had to keep moving forward, resuming my studies for a doctorate, going back to meetings, going out with my friends, driving a car again; all this, hand in hand with my family and friends, those who were new ones and those who were still with me.*

*My friends were also an important part of my adaptation, from the first moment and until now, they have been with me. When I was hospitalized for 21 days, I was never alone, they organized themselves and played a role to be with me and support me during my hospitalization. When they requested blood for the surgery, they did not hesitate at any time to donate, they are still present.*

*My family was a fundamental piece, the presence of my husband, my brothers, and my father, without a doubt lightened this process. They continued with me throughout my rehabilitation, my sister practically lived with me for a year and a half, she transferred me from one place to another; in the mornings, she took me to my job; on rehabilitation days, she was my companion; my father never lost faith, he longed to see me walking again; when he lost his battle against COVID, he left with that hope, so that’s been my legacy, to keep trying and keep persisting.*

*My husband and life partner, the first weeks were the most difficult for him, during the first three months his health was also complicated, he had a hypertensive crisis due to stress. There were days when we were both in bed, I was able to sit up and move from the bed to the chair, so I had to help him with his medication, he was more pessimistic about everything. I remember that one day he told me, “It’s that nothing is going to be the same anymore, we won’t be able to travel anymore, we won’t be able to do anything”, and, of course, he was having a negative perspective, he understood that he was speaking from ignorance of the situation, he had not seen that a person with spinal cord injury can lead a completely normal life, can travel, can take planes, can drive a vehicle, so I would have to teach him and so I did. This teaching was not only with videos, I had to teach him with my own example, so I decided, even more, to go back to everything that I had left pending before the accident, and so it is and so it will be. I re-enrolled in my doctoral studies, we have traveled multiple times, and we flew to Panama because I had to present a paper at a congress.*

*My 10-year-old daughter has been my best teacher, during these four years she was the most adapted to all this; together with me, she has learned to be independent, and she knows that women are strong. I remember that as a good mother, I worried about her mental stability and was afraid of hearing devastating answers. I asked her, daughter, what do you think about me being in a wheelchair? And her response was: Mommy is great! Because you are already my size, and I understood that I should see the positive things in the circumstance, but I understood that we would be fine.*

The concepts of the TRM that were found in the informant’s narrative are described below.

Stimuli:

Focal: Physical changes occur at the onset of a chronic disease. The severity of initial symptoms in chronic illness is the primary or focal stimulus facing the person adapting to chronic health conditions [16]: Spinal cord injury, Becoming a patient, Pain, Bowel and bladder dysfunction, Loss of control of the body, and Loss of independence for activities of daily living.

Contextual: Contextual stimuli are defined in the model as all other stimuli present that can contribute to the effect of the focal stimulus [16]: Access to rehabilitation programs, Social and family support, Being a nurse, and Living with a disability.

Residual: Residual stimuli defined as those factors with an uncertain contribution to the effect of focal stimuli. Residual stimuli may include the patient’s developmental stage and other individual factors [16]. Young Woman, Married, and Mother.

Coping strategies:

Individual adaptation processes (intrinsic).

Cognitive: Individuals must perceive changes in physical health, obtain information, and go through information processing. As the need for information grows, people seek information through established formal and informal channels [16]. Prior Nursing Knowledge.

Physiological Regulator: The regulatory subsystem involves the nervous, chemical, and endocrine systems [16]. Functional limitation.

Self-management: Self-management is the process by which the individual assumes the responsibility, tasks, and perspective necessary for the effective implementation of health care practices [16]. Personality traits, Resilience, Fortitude, and Courage.

Engage with others (extrinsic).

Seeking medical attention, nursing interventions, and Seeking social support [16]. Search for information, Search for a support group, Nursing interventions and treatments, and Seeking social support.

Enhanced Functional Capacity: Functional outcomes are a direct result of adaptation processing and influence adaptation in all modes. In the physiological mode, adaptive responses include stabilization of basic physiological balance, with a corresponding decrease in symptom severity [16]. Trunk recovery, Upper limb strength gains, Elimination control, Body recognition: recognize the pattern of symptoms, and Self-monitoring of the condition.

Results:

Normality: Normality is a key concept in adaptation in the self-concept mode. As an adaptation to chronic disease progresses, the person develops a new normal. This new state may be stable or unstable and may again require nursing assistance to achieve the best adaptive response of a person [16]. Personality, Worldview, Hope, Personal satisfaction, Recovery of role, Transformation of being, Self-control, and Confidence in the future.

**Figure 1:**
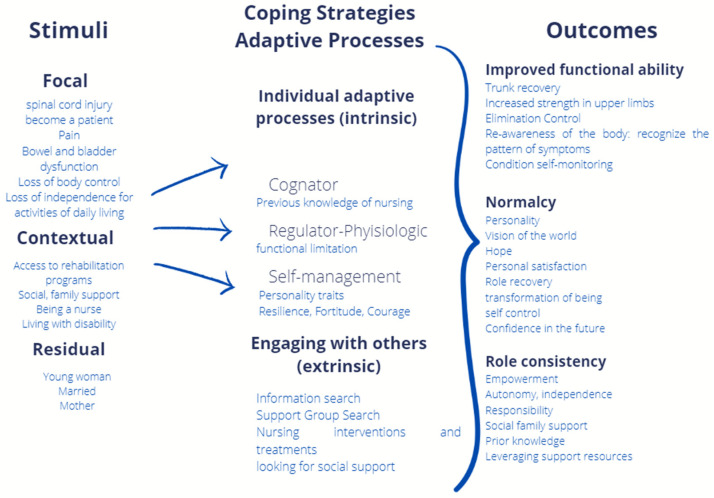
Identification of experiences in adaptation to spinal cord injury in relation to the TRM concepts of adaptation to chronic health conditions. (Fernandez-Moya, Moreno-Fergusson 2022).

Role consistency: Role consistency is a stabilization of role function and interdependence as a result of adaptation. It is based on all the processes described, as well as additional processes for the use of information and use of support [16]. Empowerment, Autonomy, Independence, responsibility, social family support, Prior knowledge, and Use of support resources (Figure 1). 

## 4. Conclusions

Narrative understood through Ricoeur’s thought illustrates that when life is overwhelming and chaotic, the tension between experienced and chronological time can be affected, and embodied sensory experience of the past can disturb present time. Conversely, embodied sensory experiences in the present tense can help clarify past and present and support chronology and narrative and bring more coherence to embodied events, experiences, reactions, and sensations. Understanding the experience from the perspective of the TRM of adaptation to chronic health conditions allows us to understand the process of adaptation to a disabling condition due to a spinal cord injury, from the perspective of a nurse who was forced to modify her role as caregiver to a role of being cared for, due to the changes in her body and her corporality due to the consequences of the injury. This narrative highlights that adaptation to a SCI is not a linear process; although, people with SCI have personal and family resources that promote adaptation, there are circumstances that alter stability, such as complications associated with sequelae, neuropathic pain, intestinal and bladder dysfunction, loss of mobility, and sensitivity below the level of the injury that causes suffering, but there were circumstances that led the informant to live and identify a transformative experience, which allowed her to discover personal characteristics about which she was not aware, as well as enhancing some that she already had present. She also found in her family and friends a great source of support to face the difficulties and adapt to her new normality; the feeling of personal satisfaction for the achievements achieved and the optimism to take on new challenges stand out.

## Data Availability

Data supporting the findings are available from the corresponding author upon reasonable request.
